# Does Metabolic Status Associate With IVF Outcomes in Women Within Similar Body Mass Index Category: Evidence From a Large Cohort Study

**DOI:** 10.1111/1753-0407.70132

**Published:** 2025-08-01

**Authors:** Lin Ding, Xiaojing Lin, Peipei Pan, Yan Li, Wei Chen, Liying He, Yunsheng Xu, Hsun‐Ming Chang, Haiyan Yang, Guiquan Wang, Liangshan Mu

**Affiliations:** ^1^ Post‐doctoral Mobile Research Station Shandong University of Traditional Chinese Medicine Jinan China; ^2^ Department of Endocrinology and Metabology The First Affiliated Hospital of Shandong First Medical University & Shandong Provincial Qianfoshan Hospital Jinan China; ^3^ Reproductive Medicine Center, the First Affiliated Hospital of Wenzhou Medical University Wenzhou China; ^4^ Reproductive Medicine Center Zhongshan Hospital, Fudan University Shanghai China; ^5^ Department of Obstetrics and Gynecology China Medical University Hospital Taichung Taiwan; ^6^ Department of Reproductive Medicine, Women and Children's Hospital School of Medicine, Xiamen University Xiamen China; ^7^ Xiamen Key Laboratory of Reproduction and Genetics Xiamen China

**Keywords:** in vitro fertilization, live birth rate, metabolically healthy obese, metabolically unhealthy obese, miscarriage rate

## Abstract

**Objectives:**

Whether diverse metabolic statuses within a similar body mass index (BMI) category associate with different in vitro fertilization (IVF) outcomes.

**Design:**

A retrospective cohort study.

**Setting:**

Wenzhou, Zhengjiang Province, China.

**Population:**

This retrospective cohort study prescreened 16 458 women who underwent their first IVF and fresh embryo transfer cycle between January 2010 and December 2021.

**Methods:**

Metabolic status was assessed using the National Cholesterol Education Program–Adult Treatment Panel III criteria. Patients were then categorized into six groups: metabolically healthy normal weight, metabolically unhealthy normal weight, metabolically healthy overweight, metabolically unhealthy overweight, metabolically healthy obese, and metabolically unhealthy obese.

**Main Outcome Measures:**

The primary outcome was live birth rate.

**Results:**

Regarding live birth, rates in normal weight women were initially lower for metabolically unhealthy normal weight versus metabolically healthy normal weight (44.6% vs. 48.6%), but this was not significant after multivariate adjustment. In obese women, live birth rates were similar between metabolically unhealthy obese and metabolically healthy obese (41.5% vs. 43.9%), with no adjusted difference. For secondary outcomes, metabolically unhealthy normal weight patients had lower biochemical pregnancy rates than metabolically healthy normal weight (OR: 0.86, 95% CI: 0.76–0.98); high blood pressure was a significant risk factor for this outcome in metabolically unhealthy normal weight (OR: 0.84, 95% CI: 0.72–0.98).

**Conclusion:**

Our findings indicated that different cardio‐metabolic risk factors but a similar BMI category may have limited adverse effects on live birth rate.

## Graphical abstract

The relationship between diverse metabolic statuses within a similar body mass index (BMI) category with different in vitro fertilization (IVF) outcomes.

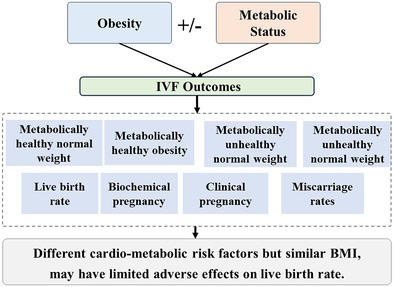

**Summary**

- Regarding live birth, rates in normal weight women were initially lower for metabolically unhealthy normal weight versus metabolically healthy normal weight, but this was not significant after multivariate adjustment.
- Blood pressure and HDL might be the most important metabolic factors influencing IVF outcomes.
- Different cardio‐metabolic risk factors but similar BMI category may have limited adverse effects on live birth rate.

## 1 Introduction

The escalating global overweight/obesity epidemic poses a significant challenge to public health worldwide. Mirroring this trend, the prevalence of obesity among adults in China surged to 14.1% in 2019 [1, 2]. This increase in obesity rates is accompanied by a corresponding rise in associated health issues, including high blood pressure, cardiovascular diseases, and even various adverse reproductive outcomes [3]. As a widely used measure of obesity, the Body Mass Index (BMI), failed to distinguish between muscle mass, bone density, fat distribution and signaling network [4], therefore BMI can both underestimate and overestimate adiposity and provide inadequate information about health at the individual level. Moreover, recent study indicated that BMI alone might covering up the phenomenon of ‘metabolic health obesity’, for the lack of consideration for other health indicators like waist circumference, blood pressure, lipid profiles, and blood glucose levels [5]. It is interesting to note that people with the same weight might be different in body composition and metabolic states. For example, normal weight obesity or the thin fat phenotype is defined as the presence of an increased body fat percentage in an individual with normal BMI [6]. Therefore, the terms “metabolically healthy overweight/obesity” and “metabolically unhealthy overweight/obesity” have been introduced to distinguish between individuals with obesity in whom cardio‐metabolic risk factors are absent or present, respectively [7]. This innovative concept not only identifies a metabolically unhealthy phenotype but also acknowledges a metabolically healthy normal weight phenotype.

Early research had established a connection between general overweight/obesity and diminished fertility, including reduced pregnancy and implantation rates, inadequate follicle development, and an elevated requirement for gonadotropin in patients undergoing in vitro fertilization (IVF) [8, 9, 10, 11]. The association between metabolically healthy overweight/metabolically healthy obese and female infertility had been explored in several studies. Our previous research identified an increased prevalence of polycystic ovary syndrome (PCOS) among metabolically healthy obese women compared to non‐obese groups [12]. In addition, a single cross‐sectional study in the National Health and Nutrition Examination Survey (NHANES) 2013–2020 database reported that individuals with metabolically healthy obese faced a higher risk of infertility compared to those with metabolically healthy normal weight [13]. The only study explored the determinants of the metabolic health status in PCOS patients with similar BMI according to metabolically healthy obese and metabolically unhealthy obese phenotypes evaluated the endocrine‐metabolic profile, inflammatory status, and body composition. Compared metabolically healthy obese with metabolically unhealthy obese patients, the latter had higher levels of worse metabolic parameters, visceral adiposity index, and fatty liver index [14]. Nevertheless, there is a scarcity of evidence concerning the relationship between different metabolic statuses and reproductive outcomes, particularly among patients with similar weight.

Therefore, we aimed to investigate the association between metabolically unhealthy/healthy phenotype within a similar BMI category and IVF outcomes based on a large retrospective cohort.

## 2 Methods

### 2.1 Research Subjects

This retrospective study utilized data extracted from the reproductive center database of The First Affiliated Hospital of Wenzhou Medical University in Wenzhou, China. Initially, a total of 16 458 infertile women completed their first IVF procedure and subsequent fresh embryo transfer cycle between January 2010 and December 2021. Exclusion criteria for this study were as follows: (i) patients aged < 20 or > 50 years (*n* = 1); (ii) patients with recurrent spontaneous abortion (RSA, *n* = 46); (iii) patients with abnormal chromosome patterns (*n* = 406); (iv) patients with abnormal renal or liver function (*n* = 39); (v) patients with thyroid dysfunction (*n* = 273); (vi) information on live birth missed (*n* = 28); (vii) body mass index (BMI) missed (*n* = 71); (viii) metabolic health status cannot be determined due to the missing data (*n* = 3473); (ix) BMI < 18.5 kg/m^2^ (*n* = 1809). Ultimately, 10 675 patients were included in subsequent analyses (Figure S1). This study received approval from the Ethics Committee of the First Affiliated Hospital of Wenzhou Medical University (No. [2023] R152).

### 2.2 Standardized Ovarian Stimulation and IVF Procedure

All patients underwent a standardized IVF‐ET procedure, encompassing ovarian stimulation, oocyte retrieval, in vitro fertilization, embryo transfer, and luteal support. The tailored ovarian stimulation protocol was based on individual factors, including ovarian function, the age of the women, infertility considerations, and adherence to center guidelines. Oocyte retrieval was conducted 34–36 h after human chorionic gonadotropin (hCG) trigger. The aspirated oocytes were fertilized by IVF, intracytoplasmic sperm injection (ICSI), or a combination of both, primarily determined by semen parameters and the couple's clinical history. Embryo morphology was observed, and fresh embryo transfer was performed 3 days after oocyte retrieval. Luteal support was initiated from the day of oocyte retrieval and continued until the 10th week of gestational age. A detailed description of the standardized IVF‐ET procedure has been previously published [15].

### 2.3 Pregnancy Outcomes

The primary outcome, live birth rate, was defined as the delivery of a viable infant at 22 weeks' gestation or beyond. Secondary outcome measures included biochemical pregnancy, clinical pregnancy, and miscarriage. Biochemical pregnancy was characterized as a serum β‐hCG level of 10 IU/L or higher detected 2 weeks post embryo transfer. Clinical pregnancy was identified by the visualization of at least one gestational sac via ultrasound. Miscarriage was classified as the termination of a pregnancy before reaching the 22nd gestational week [16].

### 2.4 Definition of Metabolic Status

The term “metabolically healthy obesity” refers to individuals with this phenotype who do not exhibit a higher risk of cardiovascular disease compared to non‐obese individuals. The classification of metabolically healthy obesity requires the simultaneous assessment of both BMI and metabolic risk factors. Participants were categorized into six mutually exclusive obesity phenotypes based on metabolic status and BMI categories: metabolically healthy normal weight, metabolically unhealthy normal weight, metabolically healthy overweight, metabolically unhealthy overweight, metabolically healthy obese, and metabolically unhealthy obese. Body mass index (BMI) was calculated as the ratio of weight in kilograms to the square of height in meters and classified according to Asian‐specific criteria [17, 18] (underweight, BMI < 18.5 kg/m^2^; normal weight, BMI of 18.5 to 22.9 kg/m^2^; overweight, BMI of 23 to 24.9 kg/m^2^; and obese, BMI ≥ 25 kg/m^2^). Metabolic status was defined using the National Cholesterol Education Program–Adult Treatment Panel III (NCEP ATP III) 2005 criteria [19] (waist circumference criterion omitted due to collinearity with BMI). Subjects were categorized as “metabolically healthy” or “metabolically unhealthy.” A person was considered “metabolically unhealthy” if presenting with two or more of the following: triglycerides level ≥ 150 mg/dL or undergoing dyslipidemia treatment; high‐density lipoprotein (HDL) cholesterol < 50 mg/dL in women; systolic blood pressure ≥ 130 mmHg, diastolic blood pressure ≥ 85 mmHg, or using antihypertensive drugs; diabetes diagnosis; fasting glucose ≥ 100 mg/dL; or receiving medications for diabetes.

### 2.5 Laboratory Testing

Blood samples were collected after a minimum of 8‐h overnight fast. Sex hormone levels were assessed utilizing an ultrasensitive enzyme‐linked immunosorbent assay (ELISA) conducted on the Unicel Dxl 800 instrument from Beckman Coulter, Brea, CA. Serum uric acid (SUA), fasting blood glucose (FBG), total cholesterol (TC), triglyceride (TG), low‐density lipoprotein (LDL), and HDL concentrations were determined using an autoanalyzer (AU 5800, Beckman Coulter). For the assessment of gonadal hormones, both intra‐assay and inter‐assay variabilities were maintained below 5%, while for other biochemical parameters, both intra‐assay and inter‐assay variabilities were kept under 10%.

### 2.6 Statistical Analysis

Discrete data were expressed as frequencies and percentages, while continuous variables were presented as means ± standard deviation (SD) or medians (interquartile ranges, IQR) depending on normality. Normality was assessed by the Shapiro–Wilk test. Differences in continuous and categorical variables among groups were assessed using one‐way ANOVA and Chi‐square tests, respectively. Separate *p* values were calculated for the metabolically healthy normal weight versus metabolically unhealthy normal weight, metabolically healthy overweight versus metabolically unhealthy overweight, and metabolically healthy obese versus metabolically unhealthy obese. Significance tests for comparison between metabolically healthy normal weight and metabolically unhealthy normal weight, metabolically healthy overweight and metabolically unhealthy overweight, as well as between metabolically healthy obese and metabolically unhealthy obese, were conducted separately.

Odds ratio (OR) and 95% confidence interval (CI) were calculated using a multivariate logistic regression model. Model 1 was unadjusted, Model 2 was adjusted for female age, and Model 3 included additional adjustments for female age, duration of infertility, fertilization methods, ovarian stimulation protocol, number of transferred embryos, basal luteinizing hormone (LH) levels, basal follicle‐stimulating hormone (FSH) levels, basal estrogen levels, and infertility factors. The duration of infertility was coded as the duration during which couples of childbearing age are unable to conceive without taking contraceptive measures and engaging in regular sexual activity. Fertilization methods included in vitro fertilization—embryo transfer or intracytoplasmic sperm injection or in vitro maturation. Ovarian stimulation protocol refers to GnRH agonist, GnRH antagonist, and mild ovarian stimulation protocol. Infertility factors refer to PCOS, endometriosis, tubal infertility factors, decreased ovarian reserve (DOR) or primary ovarian insufficiency (POI), and male infertility factors. In addition, the Rotterdam criteria have been applied for the diagnosis of individuals with PCOS [20]. According to these criteria, a patient must exhibit two of the following three symptoms: hyperandrogenism (biochemical or clinical), oligo‐ovulation or anovulation, and polycystic ovary morphology (PCOM), as determined through ultrasound examination.

Significance was determined by two‐sided *p* values less than 0.05. All analyses were performed using SAS 9.4 (SAS Institute Inc., Cary, NC).

## 3 Results

### 3.1 Baseline Characteristics of Study Population According to Metabolic Status and BMI

Among the 10 675 participants included in the final analysis, 31.3% (*n* = 3341) exhibited metabolic unhealthiness, characterized by a mean age of 31.1 ± 4.5 years and a mean BMI of 22.4 ± 2.9 kg/m^2^. The median duration of infertility was 3 (95% CI: 2–5) years, with 19.7% aged ≥ 35 years. Detailed characteristics based on healthy/unhealthy metabolic status in different groups of women are presented in Table 1. Among women with normal weight, those with metabolically unhealthy normal weight were more likely to be older, to have a longer duration of infertility, to have PCOS, and to exhibit higher basal testosterone levels compared with those with metabolically healthy normal weight (all *p* < 0.05). A similar trend was also found in women with metabolically unhealthy overweight compared with women in the metabolically healthy overweight group (all *p* ≤ 0.01). However, women in the metabolically healthy normal weight group were more likely to have higher estrogen levels (*p* < 0.01) and a younger age (*p* = 0.01) compared with those in the metabolically unhealthy normal weight group, and this trend was not significant in women with obesity (*p* = 0.24 for estrogen, *p* = 0.20 for age), but was significant in women with metabolically unhealthy overweight versus metabolically healthy overweight (*p* = 0.001 for estrogen, *p* = 0.002). Compared with metabolically healthy normal weight, metabolically unhealthy normal weight women had higher systolic blood pressure, diastolic blood pressure, and were more likely to be diagnosed with diabetes and hyperlipidemia (all *p* < 0.05), while women with obesity or overweight exhibited a similar pattern with metabolically unhealthy overweight versus metabolically healthy overweight or metabolically unhealthy obese versus metabolically healthy obese. Additionally, there was no significant difference in the type of infertility among different metabolic statuses.

**TABLE 1:** Baseline characteristics of infertile women based on body mass index and metabolic status.

Variables	Normal weight (18.5 ≤ BMI < 23 kg/m^2^)			Overweight (23 ≤ BMI < 25 kg/m^2^)			Obese (BMI ≥ 25 kg/m^2^)		*p*
	Metabolically healthy normal weight	Metabolically unhealthy normal weight	*p*	Metabolically healthy overweight	Metabolically unhealthy overweight	*p*	Metabolically healthy obese	Metabolically unhealthy obese	
*N*	5457	1486		1120	770		754	1088	
Age (year)	30.7 ± 4.3	31.2 ± 4.3	0.01	31.5 ± 4.7	32.3 ± 4.9	< 0.01	31.2 ± 4.6	31.5 ± 4.7	0.20
Infertility duration (year)	3 (2–5)	4 (2–6)	< 0.01	3 (2–5)	3 (2–6)	0.49	3 (2–5)	4 (2–6)	0.01
Infertility type, *n* (%)			< 0.01			0.34			0.32
Primary	2359 (43.2)	686 (46.2)		408 (36.4)	297 (38.6)		323 (42.8)	492 (45.2)	
Secondary	3098 (56.8)	800 (53.8)		712 (63.6)	473 (61.4)		431 (57.2)	596 (54.8)	
PCOS, *n* (%)	488 (8.9)	182 (12.3)	< 0.01	149 (13.3)	151 (19.6)	< 0.01	167 (22.2)	304 (27.9)	< 0.01
Endometriosis, *n* (%)	546 (10.0)	137 (9.2)	0.37	94 (8.4)	52 (6.8)	0.22	52 (6.9)	74 (6.8)	0.94
Tubal factor infertility, *n* (%)	3252 (59.6)	883 (59.4)	0.90	692 (61.8)	464 (60.3)	0.53	450 (59.7)	602 (55.3)	0.06
POI or DOR, *n* (%)	248 (4.5)	74 (5.0)	0.48	58 (5.2)	36 (4.7)	0.67	29 (3.9)	30 (2.8)	0.19
Male factor infertility, *n* (%)	3826 (70.1)	1131 (76.2)	< 0.01	785 (70.1)	567 (73.6)	0.10	539 (71.5)	804 (74.0)	0.24
SBP (mmHg)	110.5 ± 10.1	118.2 ± 13.6	< 0.01	113.0 ± 10.4	120.6 ± 12.4	< 0.01	114.9 ± 9.7	123.2 ± 11.9	< 0.01
DBP (mmHg)	70.5 ± 6.9	75.7 ± 8.8	< 0.01	71.6 ± 7.0	77.5 ± 8.8	< 0.01	73.3 ± 6.9	78.9 ± 8.7	< 0.01
Basal LH (IU/L)	4.6 (3.5–6.0)	4.4 (3.3–5.9)	0.12	4.2 (3.0–5.6)	4.1 (3.0–5.6)	0.63	4.0 (2.9–5.8)	4.0 (2.8–6.0)	0.72
Basal FSH (IU/L)	7.8 (6.6–9.2)	7.8 (6.5–9.3)	0.78	7.4 (6.2–8.7)	7.3 (6.0–8.6)	0.16	7.1 (6.1–8.5)	6.9 (5.9–8.2)	0.08
Basal E2 (pmol/L)	149 (105–205)	137 (95–188)	< 0.01	143.0 (99.0–193.0)	131.0 (92.0–177.0)	0.001	137 (99–193)	134 (93–185)	0.13
Basal T (nmol/L)	1.3 (0.9–1.6)	1.3 (1.0–1.7)	0.044	1.4 (1.0–1.8)	1.4 (1.0–1.7)	0.99	1.4 (1.0–1.8)	1.5 (1.1–1.9)	0.02
FBG (mmol/L)	5.2 (4.9–5.4)	5.6 (5.3–5.8)	< 0.01	5.2 (5.0–5.4)	5.6 (5.2–5.9)	< 0.01	5.2 (5.0–5.4)	5.6 (5.2–5.9)	< 0.01
TC (mmol/L)	4.5 ± 0.8	4.5 ± 0.9	0.58	4.6 ± 0.9	4.8 ± 1.0	0.0001	4.6 ± 0.8	4.8 ± 0.9	< 0.01
TG (mmol/L)	0.8 (0.6–1.1)	1.5 (0.9–2.0)	< 0.01	0.9 (0.7–1.2)	1.8 (1.1–2.3)	< 0.01	1.1 (0.8–1.4)	1.8 (1.2–2.4)	< 0.01
LDL (mmol/L)	2.4 ± 0.6	2.5 ± 0.7	< 0.01	2.6 ± 0.7	2.7 ± 0.8	0.002	2.8 ± 0.7	2.9 ± 0.7	< 0.01
HDL (mmol/L)	1.5 ± 0.3	1.2 ± 0.2	< 0.01	1.4 ± 0.3	1.2 ± 0.2	< 0.01	1.4 ± 0.2	1.1 ± 0.2	< 0.01
Ovarian stimulation protocol, *n* (%)			0.036			0.26			0.07
Agonist	4974 (91.2)	1356 (91.3)		988 (88.2)	661 (85.8)		658 (87.3)	907 (83.4)	
Antagonist	349 (6.4)	78 (5.3)		97 (8.7)	76 (9.8)		74 (9.8)	140 (12.9)	
Others	134 (2.5)	51 (3.4)		35 (3.1)	33 (4.3)		22 (2.9)	41 (3.8)	

*Note:* Data were means ± standard deviation or medians (interquartile ranges) for skewed variables or numbers (proportions) for categorical variables.

Abbreviations: BMI, body mass index; DBP, diastolic blood pressure; DOR, decreased ovarian reserve; E2, basal estrogen levels; FBG, fasting blood glucose; FSH, basal follicle‐stimulating hormone; HDL, high‐density lipoprotein cholesterol; LDL, low‐density lipoprotein; LH, basal luteinizing hormone; PCOS, polycystic ovary syndrome; POI, primary ovarian insufficiency; SBP, systolic blood pressure; T, testosterone; TC, total cholesterol; TG, triglyceride.

### 3.2 IVF Outcomes Based on Body Mass Index and Metabolic Status

Table 2 showed the reproductive outcomes on the basis of BMI and metabolic status. Patients with metabolically healthy normal weight were more likely to have more mature oocytes and embryos obtained compared with metabolically unhealthy normal weight women (all *p* < 0.05). Moreover, among women with normal weight, we observed that the rates of biochemical pregnancy, clinical pregnancy, and live birth all decreased in metabolically healthy normal weight versus metabolically unhealthy normal weight, with rates of 65% in metabolically healthy normal weight versus 61% in metabolically unhealthy normal weight for biochemical pregnancy, 58.2% in metabolically healthy normal weight versus 54.3% in metabolically unhealthy normal weight for clinical pregnancy, and 48.6% in metabolically healthy normal weight versus 44.6% in metabolically unhealthy normal weight for live birth, separately (all *p* < 0.01). However, in women with overweight, there was no difference in the rates of biochemical pregnancy, clinical pregnancy, miscarriage, or live birth between metabolically healthy overweight versus metabolically unhealthy overweight (all *p* > 0.05). Furthermore, for women with obesity, when compared those in metabolically unhealthy obese with those in metabolically healthy obese groups, only the rate of miscarriage was significantly increased from 12.0% to 17.8% (*p* = 0.01), while the rate of live birth was similar between metabolically healthy obese versus metabolically unhealthy obese (43.9% vs. 41.5%, *p* = 0.31).

**TABLE 2:** IVF outcomes based on body mass index and metabolic status.

Variables	18.5 ≤ BMI < 23 kg/m^2^			23 ≤ BMI < 25 kg/m^2^			BMI ≥ 25 g/m^2^		
	Metabolically healthy normal weight	Metabolically unhealthy normal weight	*p*	Metabolically healthy overweight	Metabolically unhealthy overweight	*p*	Metabolically healthy obese	Metabolically unhealthy obese	*p*
No. of retrieved oocytes	11 (7–15)	11 (7–14)	< 0.01	11 (7–5)	10 (6–4)	< 0.01	10 (7–14)	10 (6–13)	0.05
No. of mature oocytes	10 (6–13)	9 (6–13)	0.04	10 (6–3)	8 (5–12)	< 0.01	9 (6–12)	8 (5–12)	0.06
No. of normally fertilized zygotes	7 (4–10)	7 (4–9)	< 0.01	7 (4–10)	6 (3–9)	< 0.01	6 (4–9)	6 (3–9)	0.04
No. of embryos obtained	7 (4–9)	6 (4–9)	< 0.01	6 (4–9)	6 (3–9)	< 0.01	6 (4–8)	6 (3–8)	0.04
No. of good‐quality embryos	3 (1–5)	3 (1–5)	0.05	3 (1–5)	3 (1–5)	< 0.01	3 (1–5)	3 (1–5)	0.22
Biochemical pregnancy	3545 (65.0)	906 (61.0)	< 0.01	682 (60.9)	448 (58.2)	0.24	488 (64.7)	672 (61.8)	0.20
Clinical pregnancy	3175 (58.2)	807 (54.3)	< 0.01	611 (54.6)	404 (52.5)	0.37	417 (55.3)	599 (55.1)	0.92
Miscarriage	388 (12.4)	101 (12.8)	0.77	73 (12.3)	61 (15.6)	0.13	48 (12.0)	102 (17.8)	0.01
Live birth	2653 (48.6)	663 (44.6)	< 0.01	505 (45.1)	321 (41.7)	0.14	331 (43.9)	452 (41.5)	0.31

*Note:* Data were presented as medians (interquartile ranges) for skewed variables or numbers (proportions) for categorical variables.

Abbreviations: BMI, body mass index; IVF, in vitro fertilization.

### 3.3 Association Between Healthy/Unhealthy Metabolic Status and IVF Outcomes

Table 3 presents the ORs for different IVF outcomes based on BMI and metabolic health status, including biochemical or clinical pregnancy, miscarriage, and live birth. In normal weight women, compared with the metabolically healthy normal weight group in model 1, the risks of biochemical and clinical pregnancy were significantly lower in the metabolically unhealthy normal weight group, with the unadjusted ORs of 0.84 (95% CI: 0.75–0.95) for biochemical pregnancy and 0.85 (95% CI: 0.76–0.96) for clinical pregnancy. This trend remained significant when further adjusted for age in model 2. However, in model 3, after additional adjustments for duration of infertility, fertilization methods, ovarian stimulation protocol, number of transferred embryos, basal sex hormone levels, and infertility factors, patients in the metabolically unhealthy normal weight groups showed a lower biochemical pregnancy rate (OR = 0.86, 95% CI: 0.76–0.98), but no difference in the odds of clinical pregnancy (OR = 0.88, 95% CI: 0.78–1.00), compared to the metabolically healthy normal weight group. For the rate of live birth, compared with metabolically unhealthy normal weight, the ORs in metabolically healthy normal weight were 0.85 (95% CI: 0.76–0.96) for model 1, and 0.88 (95% CI: 0.79–0.99) for model 2 after adjustment for age. However, after adjustment for more confounding factors in model 3, no difference in the odds of live birth was found in metabolically unhealthy normal weight versus metabolically healthy normal weight. Meanwhile, there were no statistically significant differences regarding miscarriage rate in metabolically unhealthy normal weight groups compared with the metabolically healthy normal weight group in unadjusted or multi‐adjusted models.

**TABLE 3:** Associations between metabolic status and in vitro fertilization outcomes in women within similar body mass index.

In vitro fertilization outcomes	OR (95% CI), metabolically unhealthy normal weight compared to metabolically healthy normal weight	*p*	OR (95% CI), metabolically unhealthy overweight compared to metabolically healthy overweight	*p*	OR (95% CI), metabolically unhealthy obesity compared to metabolically healthy obesity	*p*
**Biochemical pregnancy**						
Model 1	0.84 (0.75–0.95)	< 0.01	0.89 (0.74–1.08)	0.24	0.88 (0.73–1.07)	0.20
Model 2	0.87 (0.77–0.98)	0.02	0.95 (0.79–1.15)	0.62	0.90 (0.74–1.09)	0.28
Model 3	0.86 (0.76–0.98)	0.02	0.94 (0.77–1.14)	0.53	0.95 (0.78–1.17)	0.65
**Clinical pregnancy**						
Model 1	0.85 (0.76–0.96)	< 0.01	0.92 (0.77–1.11)	0.37	0.99 (0.82–1.19)	0.91
Model 2	0.88 (0.79–0.99)	0.04	0.98 (0.81–1.19)	0.85	1.01 (0.84–1.22)	0.90
Model 3	0.88 (0.78–1.00)	0.046	0.96 (0.79–1.17)	0.74	1.07 (0.88–1.30)	0.45
**Miscarriage**						
Model 1	1.03 (0.82–1.31)	0.46	1.32 (0.92–1.91)	0.13	1.58 (1.09–2.29)	0.02
Model 2	1.01 (0.80–1.28)	0.42	1.27 (0.88–1.83)	0.21	**1.55 (1.07–2.24)**	**0.01**
Model 3	0.98 (0.76–1.24)	0.83	1.18 (0.81–1.73)	0.39	**1.48 (1.01–2.16)**	**0.046**
**Live birth**						
Model 1	0.85 (0.76–0.96)	0.01	0.87 (0.72–1.05)	0.14	0.91 (0.75–1.10)	0.31
Model 2	0.88 (0.79–0.99)	0.04	0.93 (0.77–1.13)	0.46	0.93 (0.77–1.12)	0.43
Model 3	0.89 (0.79–1.01)	0.06	0.96 (0.79–1.16)	0.66	0.98 (0.80–1.19)	0.82

*Note:* The bold highlighted values is the statistically significant results. Values shown as Odds ratio, OR (95% confidence interval, CI). Model 1, unadjusted; Model 2, adjusted for age; Model 3, adjusted for age, duration of infertility, fertilization methods, ovarian stimulation protocol, number of transferred embryos, basal luteinizing hormone levels, basal follicle‐stimulating hormone levels, and basal estrogen levels, and Infertility factors, such as polycystic ovary syndrome, endometriosis, tubal infertility factors, decreased ovarian reserve or primary ovarian insufficiency, and male infertility factors.

Among those with obesity, compared with metabolically healthy obese, univariate analysis demonstrated no significance for both biochemical pregnancy (OR = 0.88, 95% CI: 0.73–1.07) and clinical pregnancy (OR = 0.99, 95% CI: 0.82–1.19). After adjustment for age (model 2), the odds for biochemical pregnancy and clinical pregnancy in metabolically unhealthy obese still showed no difference. Compared with metabolically healthy obese, in model 3 after adjustment for other confounding factors, the odds remained insignificant for biochemical pregnancy (OR = 0.95, 95% CI: 0.78–1.17) and for clinical pregnancy (OR = 1.07, 95% CI: 0.88–1.30). Notably, compared with the metabolically healthy obese group in model 1, the prevalence risks of miscarriage were significantly higher in the metabolically unhealthy obese group, with the unadjusted ORs of 1.58 (95% CI: 1.09–2.29). After adjustment for age (model 2), the odds for the miscarriage rate increased (OR = 1.55, 95% CI: 1.07–2.24) in the metabolically unhealthy obese group. Moreover, with a further adjustment for confounding factors in model 3, a 48% increase in OR for miscarriage in the metabolically unhealthy obese group was found compared to the metabolically healthy obese group (OR = 1.48, 95% CI: 1.01–2.16). Finally, there were no statistically significant differences regarding live birth rate in metabolically unhealthy obese groups compared with the metabolically healthy obese group in both unadjusted and multivariable adjusted models (Table 3).

### 3.4 Association of Individual Risk Factors of Metabolic Health With IVF Outcomes

As indicated in Table 4, among those with normal weight, blood pressure emerged as having the strongest association with biochemical pregnancy (OR = 0.84, 95% CI: 0.72–0.98). Meanwhile, among obese women, the prevalence of lower HDL level exhibited a strong association with increased miscarriage rate (OR = 1.62, 95% CI: 1.06–2.47). For the rate of live birth, neither those with normal weight nor those with obesity showed a significant relationship with the metabolic risk factor used to define metabolic health. Additionally, no statistically significant associations were found between blood pressure, other lipid levels, and glucose levels, with other IVF outcomes. Further collinearity analysis of BMI as a continuous variable in the model showed no collinearity (Table S1).

**TABLE 4:** Association between individual metabolic factors and in vitro fertilization outcomes in women with similar body mass index.

	Biochemical pregnancy	Clinical pregnancy	Miscarriage	Live birth
**18.5 ≤ BMI < 23 kg/m** ^**2**^				
Triglycerides ≥ 1.7 mmol/L or medication	0.93 (0.77–1.13)	0.90 (0.75–1.08)	1.02 (0.71–1.45)	0.89 (0.74–1.07)
Blood pressure ≥ 130/85 mmHg or medication	**0.84 (0.72–0.98)**	0.86 (0.74–1.01)	0.87 (0.62–1.20)	0.90 (0.77–1.05)
Fasting glucose ≥ 5.6 mmol/L or medication	0.93 (0.83–1.05)	0.91 (0.81–1.02)	1.07 (0.85–1.34)	0.91 (0.81–1.03)
HDL (≤ 1.29 mmol/L)	0.93 (0.77–1.13)	1.01 (0.90–1.12)	0.98 (0.79–1.21)	1.00 (0.90–1.11)
**23 ≤ BMI < 25 kg/m** ^**2**^				
Triglycerides ≥ 1.7 mmol/L or medication	0.93 (0.72–1.19)	0.97 (0.76–1.24)	1.01 (0.62–1.65)	1.00 (0.78–1.28)
Blood pressure ≥ 130/85 mmHg or medication	0.96 (0.75–1.22)	0.90 (0.71–1.15)	1.17 (0.73–1.88)	0.93 (0.73–1.18)
Fasting glucose ≥ 5.6 mmol/L or medication	0.98 (0.79–1.21)	0.96 (0.78–1.18)	1.03 (0.68–1.57)	0.92 (0.74–1.13)
HDL (≤ 1.29 mmol/L)	1.08 (0.89–1.32)	1.06 (0.87–1.28)	1.04 (0.71–1.54)	1.07 (0.88–1.30)
**BMI ≥ 25 kg/m** ^**2**^				
Triglycerides ≥ 1.7 mmol/L or medication	1.17 (0.94 1.47)	1.18 (0.95–1.46)	1.09 (0.73–1.63)	1.07 (0.86–1.33)
Blood pressure ≥ 130/85 mmHg or medication	0.91 (0.73–1.13)	0.97 (0.79–1.20)	1.22 (0.83–1.80)	0.88 (0.71–1.08)
Fasting glucose ≥ 5.6 mmol/L or medication	0.95 (0.77–1.17)	1.05 (0.85–1.28)	1.11 (0.76–1.61)	1.04 (0.85–1.27)
HDL (≤ 1.29 mmol/L)	1.09 (0.88–1.35)	1.12 (0.91–1.37)	**1.62 (1.06–2.47)**	0.99 (0.80–1.22)

*Note:* The bold highlighted values is the statistically significant results. Data were presented as odds ratios (95% CI), adjusted for age, duration of infertility, fertilization methods, ovarian stimulation protocol, number of transferred embryos, basal luteinizing hormone levels, basal follicle‐stimulating hormone levels, and basal estrogen levels, and Infertility factors, such as polycystic ovary syndrome, endometriosis, tubal infertility factors, decreased ovarian reserve or primary ovarian insufficiency, and male infertility factors.

Abbreviations: BMI, Body mass index; HDL, high‐density lipoprotein.

## 4 Discussion

This study was the first investigation to date examining the collective impact of cardio‐metabolic risk factors on reproductive outcomes during IVF treatment among women within similar weight. Our findings indicated that different cardio‐metabolic risk factors but similar BMI may have limited adverse association with live birth rate. Patients with metabolically unhealthy normal weight may be less likely to achieve biochemical pregnancy compared with metabolically healthy normal weight women undergoing IVF. Metabolically unhealthy obese phenotype exhibited a higher miscarriage rate compared to those with metabolically healthy obese. Among women with normal weight, blood pressure emerged as the most significant cardio‐metabolic risk factors influencing biochemical pregnancy, while lower HDL level might increase the risk of miscarriage among obese women.

The impact of female overweight/obesity on IVF outcomes has been a subject of prolonged debate, yielding inconsistent findings. Early studies identified high maternal BMI as a significant risk factor for assisted reproductive technology failure [8, 21, 22]. A meta‐analysis confirmed a negative and significant effect of female obesity on live birth rates after IVF, indicating a decreased probability compared to normal‐weight women (Risk Ratio = 0.85, 95% CI: 0.82–0.87) [9]. However, other research had reported no differences in pregnancy or miscarriage rates between obese and non‐obese women [23, 24]. Earlier studies did not stratify results based on obesity or metabolic risk factors, potentially masking a more negative effect of increased metabolically unhealthy obesity compared to obesity defined by BMI. Additionally, conclusions from these studies were limited by unconsidered confounding factors, such as varied metabolic obesity status and predominantly European or American study populations. In China, which has the highest incidence of overweight/obese individuals, there is a lack of sufficient data on IVF outcomes in this population [25].

The concept of ‘healthy overweight/obese’ phenotype emerged in the 1980s, but significant advancements in its characterization have occurred primarily in the past decade [26]. Extensive research has been dedicated to understanding the metabolically healthy overweight/metabolically healthy obese phenotype, its associated metabolic disease risks, and mortality risks [27]. Debates have centered on whether metabolically healthy obese can be considered a “honeymoon phase” of obesity. A study by Kim et al. [28] compared the body composition, metabolic, and cardiovascular characteristics of metabolically healthy obese versus metabolically unhealthy obese in adolescent girls with PCOS. The findings revealed that adolescent metabolically unhealthy obese‐PCOS exhibited worse body composition and metabolic features compared to metabolically healthy obese‐PCOS, whether unmatched or pair‐matched for age and BMI. Another study characterized metabolically healthy obese and metabolically unhealthy obese‐PCOS patients, investigating differences in nutritional status and cardiometabolic indices in a cohort of 94 treatment‐naïve women with PCOS. The results showed that metabolically unhealthy obese‐PCOS patients had both worse endocrine and metabolic profiles, including higher levels of testosterone and elevated Homeostasis Model Assessment of Insulin Resistance (HOMA‐IR) values, compared to their metabolically healthy obese counterpart. Furthermore, metabolically unhealthy obese‐PCOS patients exhibited higher values of visceral adiposity index and fatty liver index compared to their metabolically healthy obese counterpart [14]. While early findings on the metabolic risk profile differences between metabolically healthy obese and metabolically unhealthy obese phenotypes in adult women have been proposed by several small‐sample‐sized studies, our group previously investigated the metabolic risk profile in a large survey of women with PCOS of reproductive age. The findings indicated that the metabolically unhealthy obese‐PCOS group had a worse metabolic and endocrine profile compared to the metabolically healthy obese‐PCOS group [12]. However, these studies have primarily focused on women with PCOS. In our study, consistent with previous studies, individuals with metabolically healthy obese, compared to those with metabolically healthy normal weight, exhibited higher systolic/diastolic blood pressure, glucose, and lipid levels.

Pre‐pregnancy hypertension is a well‐known risk factor for adverse pregnancy events such as preeclampsia and adverse neonatal events [29]. It is likewise well known that hypertension during pregnancy in women undergoing IVF is associated with adverse pregnancy outcomes [30]. In our study, among women with normal weight, blood pressure emerged as the most significant cardio‐metabolic risk factor influencing biochemical pregnancy. However, both systolic and diastolic blood pressure were unrelated to biochemical pregnancy, implantation, and ectopic pregnancy rate in a prospective observational cohort study based on a single IVF center at the Reproductive and Genetic Hospital of CITIC‐Xiangya [31]. Meanwhile, multivariate logistic regression analysis revealed that systolic blood pressure (OR = 0.99, 95% CI: 0.98–0.996, *p* = 0.004) and diastolic blood pressure (OR = 0.986, 95% CI: 0.98–0.998, *p* = 0.02) were negatively associated with live birth. Although the BMI of CITIC‐Xiangya in the live birth group and no‐live birth was similar (mean [SD]: 21.4 [2.4] kg/m^2^ vs. 21.5 [2.39] kg/m^2^; *p* = 0.32). The participants in CITIC‐Xiangya were different from the women in our study, which we only compared participants with normal weight (18.5 kg/m^2^ ≤ BMI ≤ 23 kg/m^2^), which might explain the difference in CITIC‐Xiangya and our study. More cohort studies with larger sample sizes should be performed in the future.

In our study for obese women, among all the metabolic factors, only low HDL level was associated with increased miscarriage rate. Steroid hormones were synthesized in thecal and granulosa cells, and cholesterol was utilized as the substrate for steroidogenesis [32]. Moreover, HDL plays important roles in ovarian cholesterol transport, and an imbalance in cholesterol homeostasis is likely to have an adverse effect on ovarian structure and function [33]. In the study of pregnant pig‐tailed macaques ( *Macaca nemestrina* ), HDL began to fall after about 4 weeks of pregnancy. Unexpectedly, the lack of an HDL decrease was reported among pregnancies that terminated in spontaneous abortions, which indicated that the normal physiologic metabolism or utilization of HDL is aberrant early in pregnancies ending in spontaneous abortions and may be due to a dysfunctional fetal‐placental unit [34]. Hongliang Wang et al. reported a tendency that mothers with higher HDL concentrations throughout gestation gave birth to infants with lower birth weight [35]. Another prospective study examined the pre‐pregnancy metabolomic profile at age 18, found a weak evidence of a positive association between total lipids and phospholipids in HDL‐cholesterol with risk of miscarriage in those women who became pregnant before 24 years old [36]. The results of the current study suggest that serum lipids, especially HDL, should be screened in women undergoing assisted reproduction.

Our study has several strengths, including a notably large sample size, a comprehensive consideration of the joint impact of female BMI and metabolic risk factors, meticulous control for confounding variables influencing IVF outcomes, and the establishment of a robust adjustment model. Notably, to the best of our knowledge, this study represents the inaugural investigation into the influence of maternal BMI on IVF outcomes. However, our research does have some limitations. Firstly, the single‐center nature of our study poses a constraint. Secondly, the growing concern regarding the impacts of insulin resistance and an unhealthy lifestyle on IVF outcomes was not specifically addressed in our study. Thirdly, it is essential to recognize that metabolic health is a transient state, and individuals transitioning from metabolically healthy status to unhealthy phenotypes across all BMI categories might be at an increased risk of various disorders. Fourthly, waist circumference is a good surrogate of central obesity; however, as a non‐routine clinical testing item, waist circumference was not directly measured in our study. Fifthly, in cohort studies, missing data is a common issue, and conducting sensitivity analysis on missing data is an important step in evaluating the robustness of research conclusions. Based on the current data, we are not capable of conducting sensitivity analysis. Therefore, future research should consider insulin resistance, lifestyles, weight change, and waist circumference of IVF patients.

## 5 Conclusion

In summary, this study revealed limited adverse associations with pregnancy outcomes in women with similar BMI but different cardio‐metabolic risk factors, as the adverse impact of metabolically unhealthy normal weight on the biochemical pregnancy and the exacerbating effects of metabolically unhealthy obese on miscarriage after IVF treatment were found. However, to solidify these findings, a larger and well‐designed study is warranted. Future research endeavors should seek to gather additional data from diverse IVF centers within the country and delve into the mechanisms underlying the influence of metabolically healthy/unhealthy obese on embryo quality and the fertilization rate.

## Author Contributions

Lin Ding, Xiaojing Lin: data curation, formal analysis, design of the manuscript, data interpretation, methodology, visualization, software, writing – original draft, writing – review and editing. Xiaojing Lin, Peipei Pan, Yan Li, Wei Chen: data curation, investigation, methodology, project administration, writing – review and editing. Liying He, Yunsheng Xu, Hsun‐Ming Chang: data interpretation, writing – review and editing. Haiyan Yang, Guiquan Wang, Liangshan Mu: conceptualization, data curation, investigation, design of the manuscript, data interpretation, methodology, project administration, resources, visualization, validation, supervision, writing – review and editing. All co‐authors contributed to critically revising the manuscript for important intellectual content and approved the final manuscript.

## Disclosure

The authors have nothing to report.

## Ethics Statement

Ethical approval was obtained from the Ethics Committee of the First Affiliated Hospital of Wenzhou Medical University (No. [2023] R152).

## Conflicts of Interest

The authors declare no conflicts of interest.

## Supporting information


**Figure S1:** Patient flow diagram.

**Table S1:** showed the associations between metabolic status and in vitro fertilization outcomes in women with similar body mass index with BMI as continuous variable.

**Table S2:** Baseline characteristics of women in included and excluded groups.

## Data Availability

The data underlying this article will be shared on reasonable request to the corresponding author.
