# Supplementary figures and images for: Does Metabolic Status Associate With IVF Outcomes in Women Within Similar Body Mass Index Category: Evidence From a Large Cohort Study
Source: J Diabetes. 2025 Aug 1;17(8):e70132. doi: 10.1111/1753-0407.70132 (PMC12317112; doi:10.1111/1753-0407.70132)

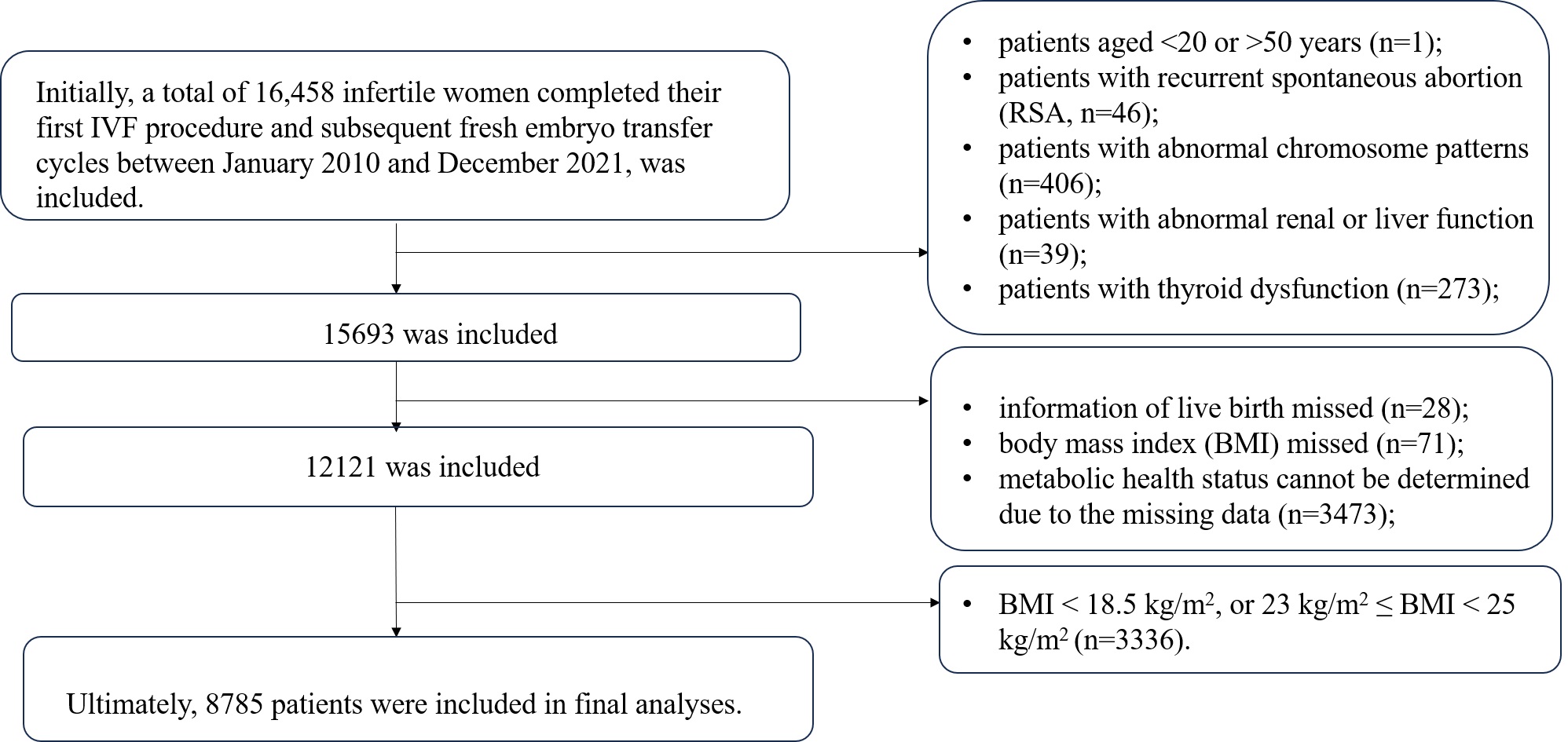

Supplement: Supplementary file 1 — Figure S1: Patient flow diagram. [file JDB-17-e70132-s003.jpg]
