# Supplementary material for: Does Metabolic Status Associate With IVF Outcomes in Women Within Similar Body Mass Index Category: Evidence From a Large Cohort Study
Source: J Diabetes. 2025 Aug 1;17(8):e70132. doi: 10.1111/1753-0407.70132 (PMC12317112; doi:10.1111/1753-0407.70132)
Supplement: Supplementary file 2 — Table S1: showed the associations between metabolic status and in vitro fertilization outcomes in women with similar body mass index with BMI as continuous variable. [file JDB-17-e70132-s002.docx]

**Supplementary Table 1. Associations between metabolic status and in vitro fertilization outcomes in women with similar body mass index with BMI as continuous variable**

| **In vitro fertilization outcomes** | **OR (95% CI),**  **metabolically unhealthy normal weight compared to metabolically healthy normal weight** | ***P* value** | **OR (95% CI),**  **metabolically unhealthy overweight compared to metabolically healthy overweight** | ***P* value** | **OR (95% CI),**  **metabolically unhealthy obesity compared to metabolically healthy obesity** | ***P* value** |
| --- | --- | --- | --- | --- | --- | --- |
| **Biochemical pregnancy** |  |  |  |  |  |  |
| BMI | 1.01(0.97-1.03) | 0.65 | 1.01(0.98-1.04) | 0.58 | 1.01(0.98-1.04) | 0.58 |
| Model 3 | 0.86(0.76-0.98) | 0.75 | 0.94(0.77-1.14) | 0.52 | 0.95(0.78-1.17) | 0.63 |
| **Clinical pregnancy** |  |  |  |  |  |  |
| BMI | 1.01(0.98-1.04) | 0.56 | 1.01(0.98-1.04) | 0.39 | 1.01(0.98-1.04) | 0.39 |
| Model 3 | 0.88(0.78-1.00) | 0.88 | 0.97(0.80-1.17) | 0.73 | 1.08(0.88-1.31) | 0.48 |
| **Miscarriage** |  |  |  |  |  |  |
| BMI | 1.03( 0.98-1.09) | 0.42 | 1.03(0.98-1.09) | 0.24 | 1.03(0.98-1.09) | 0.24 |
| Model 3 | 0.96(0.75-1.22) | 0.96 | 1.18(0.80-1.73) | 0.40 | 1.46(0.99-2.14) | 0.05 |
| **Live birth** |  |  |  |  |  |  |
| BMI | 0.998(0.97-1.03) | 0.91 | 0.998(0.97-1.03) | 0.91 | 0.998(0.97-1.03) | 0.91 |
| Model 3 | 0.89(0.79-1.01) | 0.79 | 0.96(0.79-1.17) | 0.66 | 0.98(0.80-1.20) | 0.84 |

NOTE: Values shown as Odds ratio, OR (95% confidence interval, CI)

Model 1, unadjusted; Model 2, adjusted for age; Model 3, adjusted for BMI, age, duration of infertility, fertilization methods, ovarian stimulation protocol, number of transferred embryos, basal luteinizing hormone levels, basal follicle-stimulating hormone levels, and basal estrogen levels, and Infertility factors, such as polycystic ovary syndrome, endometriosis, tubal infertility factors, decreased ovarian reserve or primary ovarian insufficiency, and male infertility factors.
