# Supplementary material for: Does Metabolic Status Associate With IVF Outcomes in Women Within Similar Body Mass Index Category: Evidence From a Large Cohort Study
Source: J Diabetes. 2025 Aug 1;17(8):e70132. doi: 10.1111/1753-0407.70132 (PMC12317112; doi:10.1111/1753-0407.70132)
Supplement: Supplementary file 3 — Table S2: Baseline characteristics of women in included and excluded groups. [file JDB-17-e70132-s001.docx]

**Supplementary Table 2. Baseline characteristics of women in included and excluded groups.**

|  | **Inlcuded groups** | **Excluded groups** |
| --- | --- | --- |
| N,% | 10675 (33.3) | 21401 (66.7) |
| Age (year) | 31.1±4.5 | 32.1±5.1 |
| Infertility duration (year) | 3.0 (2.0-5.0) | 3.0 (2.0-5.0) |
| SBP (mmHg) | 114.2±11.9 | 111.2±11.9 |
| DBP (mmHg) | 72.9±8.1 | 72.0±8.3 |
| Basal_FSH | 7.6 (6.4-9.0) | 7.6 (6.4-9.1) |
| Basal_T | 1.3 (1.0-1.7) | 1.3 (1.0-1.8) |
| TC (mmol/L) | 4.5±0.8 | 4.6±0.9 |
| TG (mmol/L) | 1.0 (0.7-1.4) | 1.0 (0.7-1.4) |
| LDL-C (mmol/L) | 2.6±0.7 | 2.6±0.7 |
| HDL-C (mmol/L) | 1.4±0.3 | 1.4±0.3 |
| PCOS, n (%) | 1441 (13.5) | 2882 (13.5) |

NOTE: Data were means ± standard deviation or medians (interquartile ranges) for skewed variables or numbers (proportions) for categorical variables. systolic blood pressure (SBP), diastolic blood pressure (DBP), basal follicle-stimulating hormone (FSH), testosterone, (T), polycystic ovary syndrome (PCOS), total cholesterol (TC), triglyceride (TG), low-density lipoprotein (LDL), high-density lipoprotein cholesterol (HDL).
